# Ginsenoside Compound K Regulates HIF-1α-Mediated Glycolysis Through Bclaf1 to Inhibit the Proliferation of Human Liver Cancer Cells

**DOI:** 10.3389/fphar.2020.583334

**Published:** 2020-12-08

**Authors:** Silin Zhang, Meilan Zhang, Jiaxin Chen, Jiaqi Zhao, Jielin Su, Xuewu Zhang

**Affiliations:** ^1^ College of Medicine, Yanbian University, Yanji, China; ^2^ Yanbian University Hospital, Yanji, China

**Keywords:** HIF-1α, glycolysis, liver cancer, compound K, Bclaf1

## Abstract

This study aimed to demonstrate that ginsenoside compound K (20 (S)-ginsenoside CK; CK) downregulates Bcl-2-associated transcription factor 1 (Bclaf1), which inhibits the hypoxia-inducible factor-1α (HIF-1α)-mediated glycolysis pathway to inhibit the proliferation of liver cancer cells. Treatment of hepatoma cells (Bel-7404 and Huh7) under hypoxic conditions with different concentrations of CK showed that CK inhibited the proliferation of hepatoma cells in a time- and concentration-dependent manner; furthermore, the ability of the cells to form colonies was reduced, and cell growth was blocked in the G0/G1 phase. CK promoted the degradation of HIF-1α ubiquitination in liver cancer cells by regulating the expression of HIF-1α and related ubiquitination proteins; moreover, it reduced the activity of key enzymes involved in glycolysis, the pressure of cellular glycolysis, and the rate of real-time ATP production, thereby inhibiting the glycolysis pathway. It also decreased the expression of Bclaf1 in hypoxic liver cancer cells and thus reduced the ability of Bclaf1 to bind to HIF-1α. CK treatment of Bel-7404 and Huh7 cells with CRISPR/Cas9-engineered knock out of Bclaf1 gene under hypoxic conditions further suppressed the expression of HIF-1α, promoted HIF-1α ubiquitination, and inhibited the glycolysis pathway. In a rat model of primary liver cancer induced by diethylnitrosamine, positron emission tomography and computed tomography scans showed that after CK administration, tumor tissue volumes were reduced and glucose uptake capacity decreased. Increased Bclaf1 and HIF-1α expression promoted the ubiquitination of HIF-1α and inhibited the glycolysis pathway, thereby inhibiting the proliferation of liver cancer cells. In summary, this study confirmed by *in vitro* and *in vivo* experiments that in hypoxic liver cancer cells CK downregulates the expression of Bclaf1, inhibits the HIF-1α-mediated glycolysis pathway, and inhibits cell proliferation, suggesting that the CK-mediated effects on Bclaf1 may represent a novel therapeutic approach for the treatment of liver cancer patients.

## Introduction

Primary liver cancer is among the most common malignant tumors, with the sixth highest incidence and second highest mortality rate worldwide (Gao et al., 2017; Shen et al., 2017). Hepatocellular carcinoma is the most common type of primary liver cancer. Treatments for hepatocellular carcinoma include surgical resection, radiation therapy, chemotherapy, and liver transplantation. However, owing to the non-specific symptoms of liver cancer, most cases are diagnosed in the middle and late stages of the disease. Treatment for patients with advanced liver cancer is limited and prone to drug resistance (Bhayani et al., 2015). Therefore, there is a need for new chemotherapy drugs or targeted therapies.

Ginseng is an essential medicinal plant with widespread application in Asian countries and, in particular, in traditional Chinese medicine (TCM). Natural products or herbal medicines are commonly used in traditional medicine practice for cancer treatment, including liver cancer (Ling et al., 2018; Tang et al., 2020). Ginsenoside is the main component of ginseng and exerts many pharmacological activities, including anti-inflammatory, anti-tumor, anti-dementia, and anti-allergic effects (Park et al., 2003; Nag et al., 2012; Shi et al., 2013). Ginsenoside compound K (CK) is a ginsengdiol-type saponin derived from ginsenosides Rb1, Rb2, and Rc. As a degradation product of human intestinal bacteria, it has various pharmacological activities, including liver protective, anti-inflammatory, and anti-tumor effects (Lee et al., 2005; Kim et al., 2009; Park et al., 2011). A previous study showed that CK regulated STAT3 to induce endoplasmic reticulum stress in human hepatoma cells, promoting hepatoma cell apoptosis and inhibiting proliferation (Zhang et al., 2018).

Bcl-2-associated transcription factor 1 (Bclafl) is a transcription inhibitor that regulates the transcription of the Bcl-2 gene. It has an arginine-serine domain and a DNA-binding domain and is a multifunctional protein with low expression levels in multiple tumor cell lines (Kasof et al., 1999). Bclaf1 regulates gene transcription and post-transcriptional processing, including control of lytic infection, DNA damage and repair, and post-transcriptional events such as pre-mRNA splicing and mRNA processing (Cuconati and White, 2002; Fontoura et al., 2005; Savage et al., 2014; Shao et al., 2016). In 2019, Bclaf1 was first reported to be involved in the occurrence and development of liver cancer, and was found to be highly expressed in human hepatocellular carcinoma Huh7 cells (Zhou et al., 2019). Proteomics research by the same group also found that Bclaf1 was highly expressed in human hepatocellular carcinoma tissues and compared with adjacent tissues, the difference was significant. Wen et al. (Wen et al., 2019) studied the expression levels of Bclaf1 and hypoxia-inducible factor-1α (HIF-1α) in liver cancer tissues from 473 hepatocellular carcinoma patients. Expression levels of HIF-1α in human liver cancer cells decreased after Bclaf1 knockout in a 1% O_2_ environment and significantly increased after Bclaf1 overexpression; furthermore, Anwen Shao et al. (Shao et al., 2020) reported Bclaf1 was a direct transcriptional target of HIF-1 and upregulated in multiple cell lines during hypoxia. And Bclaf1 was involved in the stabilization of HIF-1α during long-term hypoxic treatments. Compared with the control cells, the protein level and stability of HIF-1α in Bclaf1 knockdown or knockout cells is greatly compromised after long-term hypoxic treatments, concomitant with the impaired inductions of HIF-1 target gene transcription.

The rapid proliferation of liver cancer cells needs to consume a lot of oxygen, forming a local hypoxia micro-environment. Hypoxia is one of the main characteristics of hepatocellular carcinoma and plays an important regulatory role in the occurrence and development of hepatocellular carcinoma. In order for tumor cells to resist hypoxia, more than half of the ATP production method is converted from oxidative phosphorylation to glycolysis that does not require oxygen. The energy metabolism mode of glycolysis can improve the tolerance of tumor cells to ischemia and hypoxia, and promote the infiltration and metastasis of cancer cells (Cha et al., 2015). HIF-1α is an important transcription factor whose expression is induced under hypoxic conditions. It is involved in mediating cellular responses to hypoxia by promoting the expression of key glycolytic enzymes HK2, GLUT1, LDHA, and PDK1, and regulating the transcription of key enzyme target genes (Dayan et al., 2006; Masoud et al., 2015). HIF-1α affects the energy metabolism and proliferation of tumor cells through ubiquitin-proteasome degradation. Under normoxic conditions, HIF-1α ubiquitination occurs via the proline-4-hydroxylase (PHD)/von Hippel-Lindau E3 ubiquitin ligase (pVHL) pathway. After HIF-1α is hydroxylated by PHD, it is recognized and bound by pVHL and degraded by the 26S proteasome (Andersen et al., 2011; Yonashiro et al., 2018). However, PHD is oxygen dependent and its activity decreases under hypoxic conditions, and thus HIF-1α cannot undergo hydroxylation. This affects the degradation of HIF-1α via the PHD/pVHL ubiquitination pathway, resulting in the aggregation of HIF-1α in cells and binding to HIF-1β to generate stable HIF-1 dimers. Combined with homologous enhancer sequence hypoxia response elements, this dimer induces the expression of tumor-related factors and participates in tumor metastasis, angiogenesis, and metabolism (Miyake et al., 2013; Rigiracciolo et al., 2015; Wan et al., 2017). Under hypoxic conditions, heat shock proteins (HSPs) participate in the ubiquitination degradation of HIF-1α. HSP70 combines with ubiquitin ligase CHIP to promote HIF-1α degradation; when HSP70 is lacking, HSP90β competes with receptor protein kinase C1 (RACK1) to bind to HIF-1α and, after participating in the stabilization of HIF-1α, promotes its degradation by ubiquitination (Liu et al., 2007; Luo et al., 2010).

The purpose of this study was to elucidate the mechanisms involved in human hepatoma cells response to hypoxic conditions *in vivo* an *in vitro*. In the diethylnitrosamine (DEN)-induced primary hepatocellular carcinoma rat model, CK downregulated Bclaf1 expression, inhibited the HIF-1α-mediated glycolysis pathway, and affected the proliferation of hepatoma cells.

## Materials and Methods

### Reagents

**FIGURE 1:**
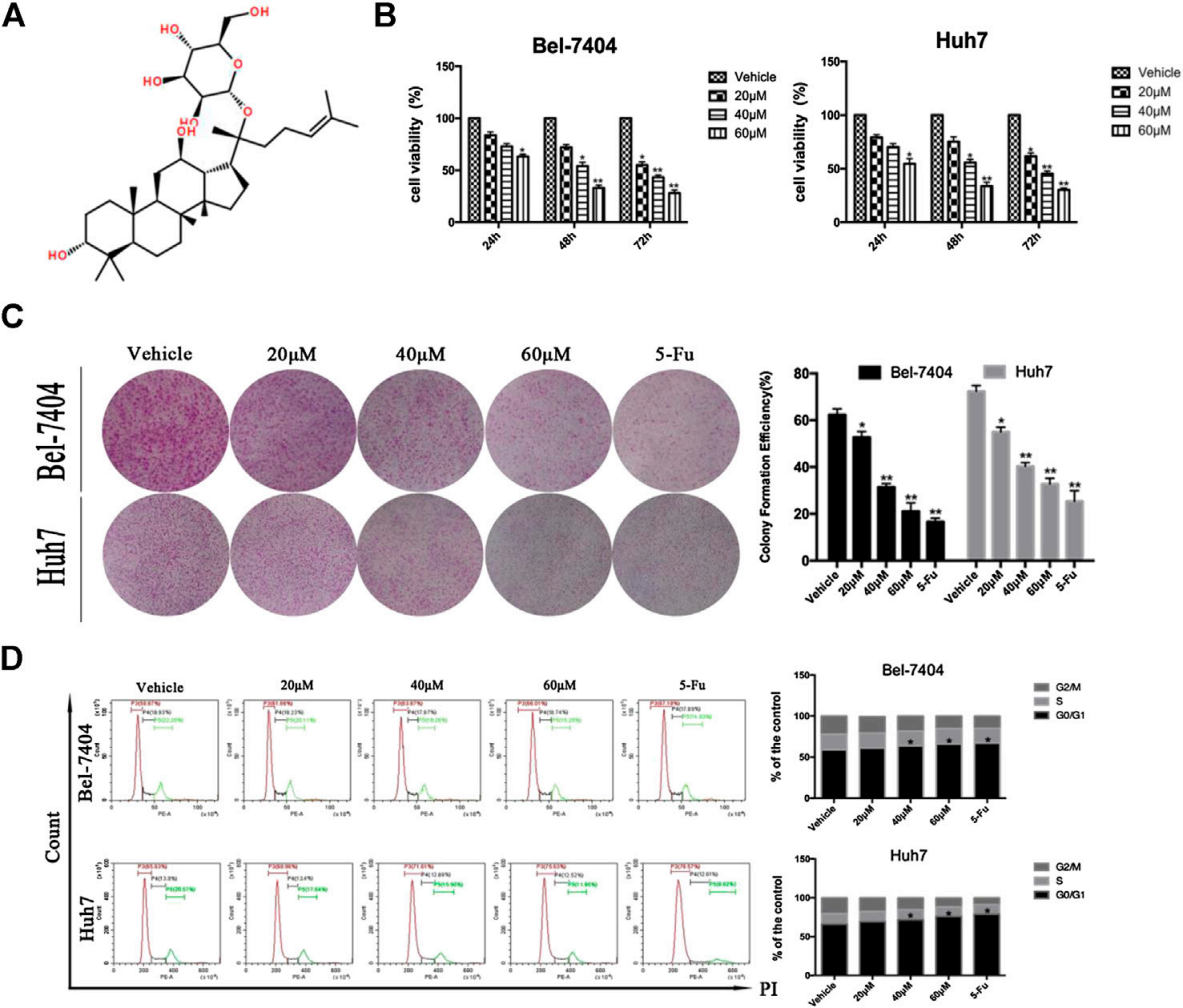
CK inhibited the proliferation of liver cancer cells under hypoxic conditions. **(A)** The chemical structural formula of CK. Bel-7404 and Huh7 cells were treated with different concentrations of CK (0, 20, 40, and 60 μM) under hypoxia for 24, 48, and 72 h **(B)** CCK8 was used to detect the viability of both cell lines. Compared with the vehicle group, the cell viability of the 40, and 60 μM CK-treated groups were significantly reduced (*p < 0.05, **p < 0.01). **(C)** Staining with crystal violet, and observing the colony formation rate of the two cell lines after 48 h of CK treatment. Colony formation rates of each group: blank control group, 20 μM > 40 μM > 60 μM>5-FU; all differences were statistically significant (*p* < 0.05). With increasing drug concentrations, the cell cluster became smaller and the number of colonies was fewer. Formation rate = (number of colonies formed /number of seeded cells) × 100%, (*p < 0.05, **p < 0.01). **(D)** After propidium iodide single staining, the cell cycle phase was analyzed by flow cytometry. In Bel-7404 cells, with increased drug concentration, the proportion of G0/G1 phase was 58.87%, 61.66%, 63.87%, 66.01%, and 67.18%; the proportion of S phase was 18.93%, 18.23%, 17.87%, 18.74%, and 17.89%; the proportion of G2/M phase was 22.20%, 20.11%, 18.26%, 15.25%, and 14.93%. The proportion of G0/G1 in Huh7 cells was 65.63%, 68.96%, 71.61%, 75.63%, and 78.57%; the proportion of S phase was 13.8%, 13.4%, 12.89%, 12.52%, and 12.61%; the proportion of G2/M phase was 20.57%, 17.64%, 15.50%, 11.85%, and 8.82%. Cell cycle analysis by flow cytometry showed significantly increased numbers of cells in the G0/G1 phase (*p < 0.05, **p < 0.01).

CK (Figure 1A) (purity 98%) and 5-fluorouracil(5-Fu) were purchased from Shanghai Yuanye Bio-Technology Co., Ltd. (Shanghai, China). Dulbecco’s modified Eagle’s medium (DMEM) and penicillin-streptomycin were purchased from Gibco-BRL (Grand Island, NY, USA), and fetal bovine serum (FBS) was obtained from BI. Rabbit-polyclonal antibodies against Bclaf1, HIF-1α, LDHA, PHD, and RACK1 were purchased from Abcam (Cambridge, MA, USA). Human-polyclonal antibody against HIF-1α was purchased from Abcam (Cambridge, MA, USA). Rabbit-polyclonal antibodies against Glut1, PDK1, HK2, HSP70, HSP90, and β-actin were purchased from Wanleibio (Shenyang, China). Secondary antibodies were purchased from ZSGB-Bio Co., Ltd. (Beijing, China). The CCK8 assay was obtained from Dojindo (Tokyo, Japan) and the cell cycle analysis kit was from Baihao (Shenyang, China).

### Cell Culture and Growth Assay

Human hepatoma cell lines (HepG2, SMMC-7721, Bel-7404, and Huh7) were purchased from Key GEN Co., Ltd. (Nanjing, China) and cultured in DMEM with 10% FBS and 100 U/mL penicillin-streptomycin at 37°C in a humidified (5% CO_2_, 95% air) incubator or in a hypoxic (1% O_2_, 5% CO_2_, 94% N_2_) chamber.

### Cell Proliferation Assessment by CCK-8 Assay

Cell proliferation was assessed using a CCK-8 Assay (Dojindo). To evaluate the antiproliferative effects of CK on human Bel-7404 and Huh7 cells, cell suspensions (8 × 10^4^/ml) were seeded in 96-well plates with growth medium overnight. Cells were treated with various concentrations of CK (20, 40, 60, and 80 µM), with 0.1% DMSO as a control. At appropriate time points (24, 48, and 72 h), 90 µL fresh medium was incubated with 10 µL CCK-8 solution in each well for 2 h at 37°C, and the absorbance was read at the 450 nm wavelength using a microplate reader (Bio-Tek, San Jose, CA, USA).

### Cell Cycle Analysis

Cell cycling was analyzed using a cell cycle analysis kit (Beyotime) according to the kit instructions. The percentage of cells in each phase of the cell cycle were determined with a CytoFLEX flow cytometer (Beckman Coulter, Inc., CA, USA) and analyzed using CyExpert software (Beckman Coulter Inc., CA, USA).

### Immunocytochemistry Staining Assay

Cells were seeded in six-well plates (1 × 10^5^ cells/well) and cultured overnight under hypoxic conditions, then treated with CK (20, 40, and 60 μM) and 5-Fu for 48 h. Briefly, Huh7 and Bel-7404 cells were blocked in 3% hydrogen peroxide for 15 min, then incubated with primary antibodies against Bclaf1 (1:100) overnight at 4°C. After washing with PBS, the cells were incubated with secondary antibodies for 1 h at 37°C. Then, the cells were stained with 3,3′-diaminobenzidine, counter-stained with hematoxylin, and photographed under a light microscope (Olympus, Tokyo, Japan) at 400× magnification. The results were assessed quantitatively via digital image analysis using ImagePro-Plus (Media Cybernetics, Silver Spring, MD, USA).

### Immunohistochemistry Staining Assay

Human liver cancer tissue samples was embedded in paraffin, sectioned, and rehydrated for immunohistochemistry staining (IHC). After antigen retrieval, sections were incubated in 3% hydrogen peroxide for 10 min. Then, the sections were blocked with goat serum for 30 min and incubated with primary antibody Bclaf1 (1:250) overnight at 4°C. The next day, the sections were incubated with secondary antibodies for 1 h at 37°C. They were then stained with 3,3′-diaminobenzidine and counter-stained with hematoxylin, before being photographed under a light microscope (Olympus, Japan) at 400 × magnification.

### Immunofluorescence Assay

Cells were treated with the indicated amounts of CK for 48 h, collected, and subjected to standard immunofluorescence (IF) assays using a confocal laser scanning microscope (Olympus, Japan) at 800× magnification.

### Co-immunoprecipitation

Cells were harvested and lysed by sonication with a Diagenode Bioruptor UCD-200 in cell lysis buffer (50 mM Tris-HCl, pH 7.5, 150 mM NaCl, 1% Nonidet P-40, 0.5% sodium deoxycholate, and 1% protease inhibitor cocktails; Sigma-Aldrich) for co-immunoprecipitation analysis (Co-IP). Cell lysates were centrifuged, and the supernatant was incubated with the appropriate antibodies and Protein G Plus/Protein A agarose beads (Calbiochem) or FLAG M2-conjugated agarose beads (Sigma-Aldrich) at 4°C overnight. The beads were washed six times with cell lysis buffer, and the precipitated proteins were further analyzed. For western blotting, equal amounts of protein (80–100 µg) from cell lysates were denatured in sample buffer (Thermo Fisher Scientific), subjected to sodium dodecyl sulfate polyacrylamide gel electrophoresis (SDS-PAGE; Bio-Rad), and transferred to nitrocellulose membranes (Thermo Fisher Scientific). The membranes were blocked in 1 × tris buffered saline Tween (TBST) with 5% milk and immunoblotted with the appropriate primary antibodies at 4°C overnight, followed by incubation at room temperature for 1 h with horseradish peroxidase-conjugated secondary antibodies. Bands were developed using an enhanced chemiluminescence (ECL) kit (Millipore, Billerica, MA, USA).

### Western Blotting

After treatment of Bel-7404 and Huh7 cells with different concentrations of CK, total protein was extracted using RIPA lysis buffer (Solarbio, Beijing, China). Proteins were separated by SDS-PAGE (100 V, 120 min) and transferred to polyvinylidene fluoride membranes (100 V, 30–90 min). Subsequently, the membranes were blocked with skimmed milk (5%) and incubated overnight at 4°C with primary antibodies (1:1000). The next day, the membranes were incubated with anti-rabbit secondary antibody (1:5000) for 2 h at room temperature. Finally, after ECL incubation, the target proteins were examined using a BIO-RAD imaging system (BIO-RAD, Hercules, CA, USA).

### CRISPR/Cas9-Mediated Bclaf1 Knockout

CRISPR-Cas9 small guide RNA (sgRNA) lentiviral vectors targeting human Bclaf1 and HIF-1α were purchased from Shanghai GeneChem Co., Ltd. (Shanghai, China). Huh7 and Bel-7404 cells were seeded in six-well plates (3 × 10^4^ cells/well) in 2 ml media and cultured overnight. The cells were treated with 25 L lentiviral vector (4 × 10^8^ TU/mL) in 1 ml media with 5 g/ml Polybrene transfection reagent. After 24 h, the media were changed for conventional culture medium and cells were continuously cultured for another 24 h. Then, 6 g/ml puromycin was used to screen transfected cells. The expression of Bclaf1 and HIF-1α was confirmed by western blot.

### Cell Glycolysis Pressure Test and Real-Time ATP Assay

Cellular glycolytic capacity was measured using a Seahorse Bioscience XF96 Extracellular Flux Analyzer, according to the instructions of the Seahorse XF Glycolysis Stress Test Kit and Seahorse XF Real-Time ATP Rate Assay Kit.

### Rat Primary Liver Cancer Model and Treatment of CK

The experimental procedures were approved by the Animal Care and Use Committee of Yanbian University and were in accordance with the animal welfare guidelines of the U. S. National Institutes of Health. The permit number was SCXK (Ji) 2011-006. Fifty-four 3-week-old clean-grade SD rats were randomly divided into an experimental group (45 rats) and control group (15 rats) after stable feeding for 7 days. Rats in the experimental group were fed with 0.01% DEN solution for 20 weeks as follows: 0–5 weeks with DEN solution, 6–8 weeks with sterilized water, 9–14 weeks with DEN solution, 14–20 weeks with sterilized water. The control group was fed with sterilized water throughout the course. After successful modeling, rats in the experimental group were injected intraperitoneally with CK (low-dose group, 2.5 mg/kg; high-dose group, 5 mg/kg), and 5-Fu (5 mg/kg) was injected intraperitoneally into rats in the positive control group. In the control group, an equal volume of normal saline was intraperitoneally injected into the rats for 2 weeks.

### Positron Emission Tomography/Computed Tomography Imaging

Rats were fasted and allowed only water for 12 h, and then were placed in an anesthesia box and 2% isoflurane in pure oxygen was administered by inhalation. After the rats were anesthetized, approximately 37 MBq of radioactive tracer 18F-FDG was injected into the tail vein. After about 35 min, the rats were positioned on the scanning bed to fully expose the liver. During positron emission tomography (PET) acquisition, computed tomography (CT) was used for attenuation correction simultaneously (PET-CT): CT correction scan for 5 min, and PET emission scan for 10 min. The scanning parameters were: voltage 80 kV, current 500 μA. During the PET image acquisition, 2% isoflurane in pure oxygen was continuously given to maintain anesthesia. The Inveon Research Workplace was used for image acquisition and Siemens software was used to analyze the standard uptake value (SUV) value of the region of interest for image analysis.

### Tandem Mass Tags Marker Quantitative Proteomics

Tissue pellets were added to the appropriate amount of SDT lysate (4% SDS, 1 mM DTT, 100 mM Tris-HCl, pH7.6), transferred to Lysing Matrix A tubes, and homogenized with an MP homogenizer (24 × 2, 6.0 M/S, 60 s, twice). After sonication, samples were boiled in a water bath for 10 min. After centrifugation at 14,000 g for 15 min, the supernatant was filtered through a 0.22 μm centrifuge tube and the filtrate was collected. Protein quantification was performed using the BCA method. The samples were dispensed and stored at −80°C. Then, 20 μg of each sample was added to 6× loading buffer, boiled in water for 5 min, subjected to 12% SDS-PAGE electrophoresis (constant pressure 250 V, 40 min), and stained with Coomassie Brilliant Blue. Finally, peptides were pre-fractionated by strong cation exchange using a Waters 600E high-performance liquid chromatography system. The resulting fractions were separated by reverse-phase chromatography using an Eksigent (Redwood City, CA) NanoLC-Ultra-2D Plus system. Mass spectra were acquired on an AB Sciex 5600 triple time-of-flight mass spectrometer.

### Statistical Analysis

All experiments were repeated three times. Data are expressed as mean ± standard deviation (SD), and differences between groups were analyzed by one-way analysis of variance and Student’s t-test. *p* < 0.05 was considered to be statistically significant. SPSS version 19.0 and GraphPad Prism 5.0 software were used to analyze the results.

## Results

### CK Inhibited the Proliferation of Hypoxic Liver Cancer Cells

The ginsenoside CK (20-O-D-glucopyranosyl 20(S)-protopanaxadiol) is a metabolite of the natural glycol-type ginsenoside metabolized by intestine intestinal bacteria. Its structural form is shown in Figure 1A. We investigated the effects of CK on the proliferation of hepatoma cells under hypoxic conditions. CK (20, 40, and 60 μM) was used to treat Bel-7404 and Huh7 cells under hypoxic conditions for 24, 48, and 72 h. Cell viability was determined by CCK8 assays. The half maximal inhibitory concentration values of CK in Bel-7404 cells were 63.78, 38.52, and 28.88 μM at 24, 48, and 72 h, respectively, and those in Huh7 cells at 24, 48, and 72 h were 64.00, 38.54, and 28.31 μM, respectively. Choosing 48 h as the CK treatment duration, the proliferation inhibition rates of Bel-7404 cells were 0%, 30.57%, 48.13%, and 64.16%, and those of Huh7 cells were 0, 19.43%, 45.5% and 64.8%, with CK doses of 20, 40, and 60 μM, respectively (Figure 1B). The ability of cells to form colonies was detected by plate colony formation assays. After 7 days of culture with fresh culture medium, the number of colonies increased compared with the vehicle group. In the CK-treated group, the number of colonies decreased and cell masses became smaller (Figure 1C). Cell cycle analysis by flow cytometry showed significantly increased numbers of cells in the G0/G1 phase, with a corresponding decrease in the G2/M phase population. However, there was no significant effect on the number of cells in the S phase (Figure 1D). These results showed that CK inhibited the proliferation of hepatocellular carcinoma cells in a dose-dependent manner. Furthermore, cells were blocked in the G0/G1 phase and could not enter the S phase, decreasing the proliferation index. Thus, CK could inhibit the growth and proliferation of hypoxic liver cancer cells.

### CK Inhibited the Expression of Bclaf1 in Hypoxic Liver Cancer Cells and the Binding of Bclaf1 to HIF-1α

**FIGURE 2:**
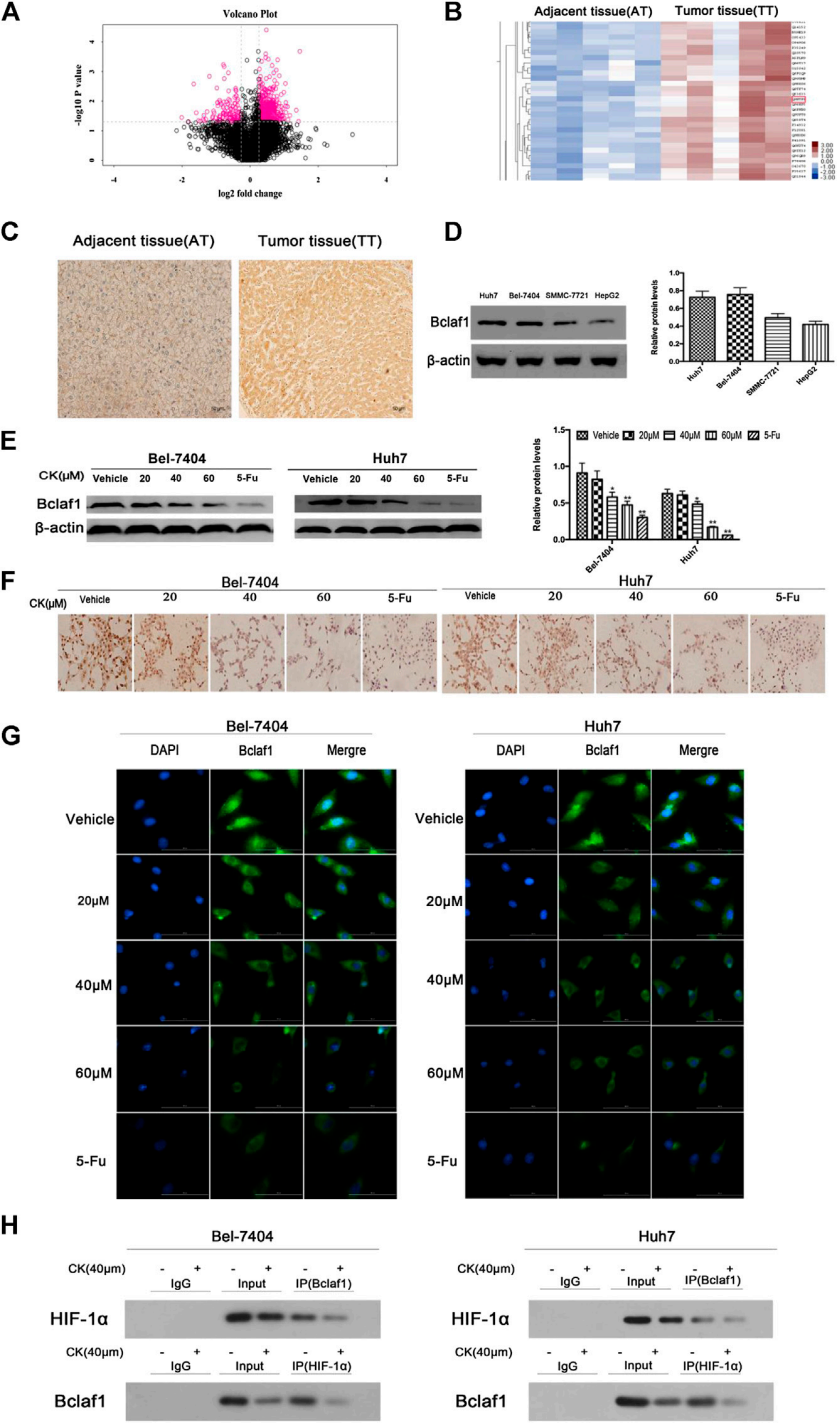
Bclaf1 expression in human liver cancer tissues and cells and its interaction with HIF-1α. **(A)** Volcano plot of differentially expressed proteins in liver tissue. **(B)** Bclaf1 is differentially expressed in liver cancer vs. adjacent tissues. **(C)** Immunohistochemical staining showed the expression and localization of Bclaf1 in liver cancer tissues. Bclaf1 is brown-yellow in both the nucleus and cytoplasm. **(D)** Expression of Bclaf1 in various human liver cancer cell lines. **(E)** After treating Bel-7404 and Huh7 cells with different concentrations of CK (0, 20, 40, and 60 μM) for 48 h under hypoxic conditions, the expression and quantitative analysis of Bclaf1 were detected by western blot. Data are expressed as mean ± standard deviation; compared with vehicle, *p < 0.05 and **p < 0.01. **(F)** Immunocytochemistry staining of Bclaf1 expression and localization by light microscopy (mag: 400×). **(G)** Immunofluorescence staining of Bclaf1 expression and localization by fluorescence microscopy (mag: 800×). **(H)** Co-immunoprecipitation analysis of the Bclaf1 and HIF- 1α binding strength.

In order to investigate whether CK could inhibit the expression of Bclaf1 and the binding of Bclaf1 to HIF-1α, we first observed the expression of Bclaf1 in human liver cancer tissues. Ten human liver cancer tissues were selected for proteomics analysis (Figure 2A), which revealed a high expression Bclaf1 protein (Figure 2B). IHC experiments showed that Bclaf1 was highly expressed in human liver cancer tissues compared with adjacent normal tissues; the difference in expression was significant (Figure 2C), and Bclaf1 was expressed in both the cytoplasm and the nucleus. In addition, Bclaf1 was expressed to varying degrees in several liver cancer cell types (Huh7, Bel-7404, SMMC-77721, and HepG2), with higher expression in Huh7 and Bel-7404 cells (Figure 2D). In order to investigate whether CK could inhibit the expression of Bclaf1 and the binding of Bclaf1 to HIF-1α, we used different concentrations of CK (20, 40, and 60 µM) to treat Bel-7404 and Huh7 cells under hypoxic conditions for 48 h. Western blot results revealed that the expression of Bclaf1 decreased (Figure 2E), IHC and IF staining showed that the expression of Bclaf1 in the nucleus and cytoplasm decreased with increasing CK concentration (Figures 2F, G). The Co-IP results showed that CK inhibited the interaction between Bclaf1 and HIF-1α (Figure 2H). This suggests that CK inhibits the expression of Bclaf1 and the binding of Bclaf1 to HIF-1α in hypoxic liver cancer cells.

### CK Promotes the Ubiquitination and Degradation of HIF-1α in Hypoxic Hepatoma Cells

**FIGURE 3:**
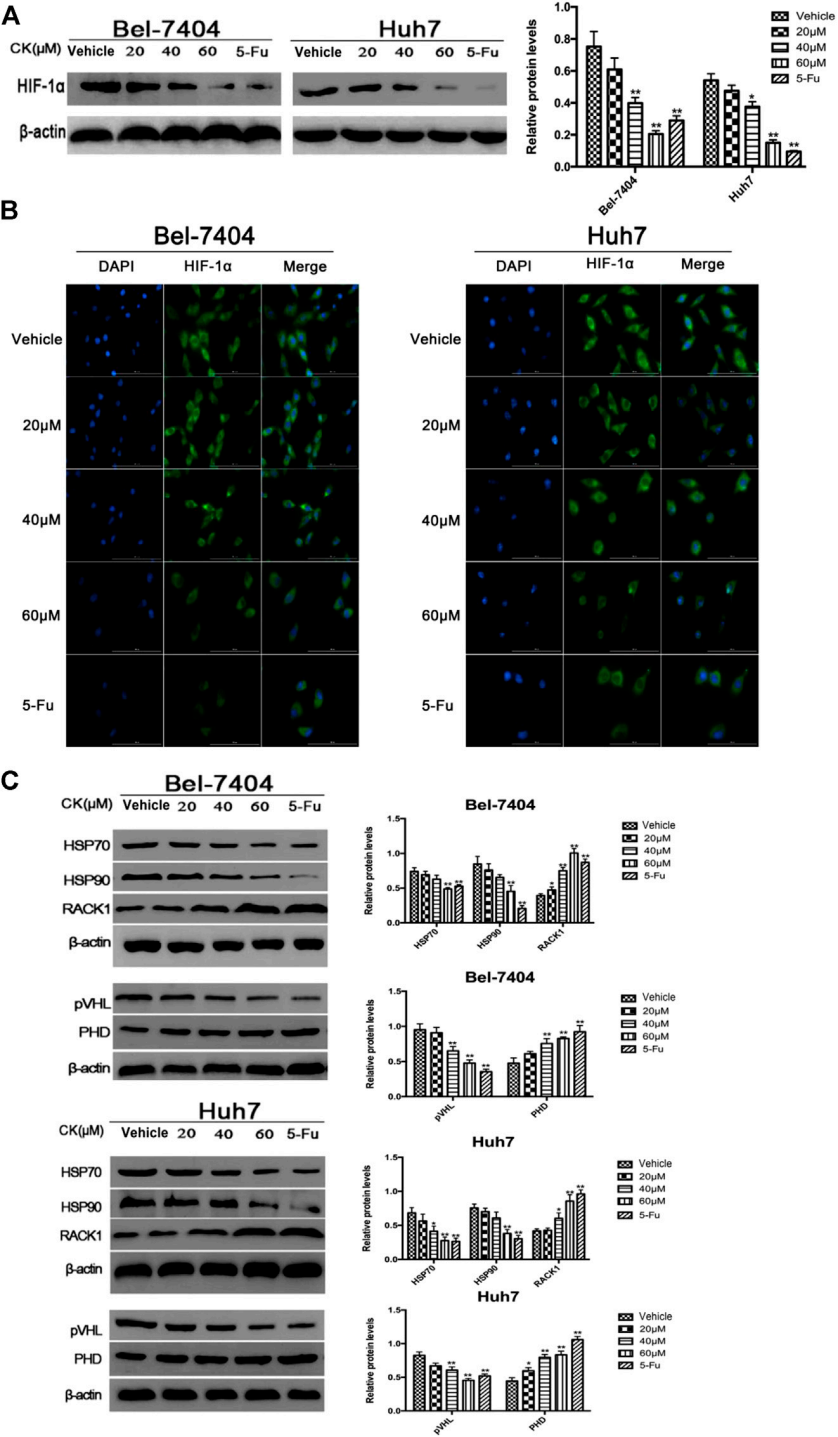
CK promotes the ubiquitination degradation of HIF-1α protein in hypoxic liver cancer cells. Treatment of hypoxic liver cancer cells Bel-7404 and Huh7 with different concentrations of CK (20, 40, and 60 μM) for 48 h: **(A)** Western blot detection of HIF-1α Expression and quantitative analysis. **(B)** After IF staining, the expression and localization of Bclaf1 (800×) were observed with a fluorescence microscope. **(C)** Western blot detection of HIF-1α ubiquitination-related proteins (HSP70, HSP90, RACK1) and (pVHL, PHD) expression and quantitative analysis, compared with vehicle group, *p < 0.05, **p < 0.01.

Glycolysis is closely related to the ubiquitination and degradation of HIF-1α (Ryan et al., 2000). To investigate whether CK affected glycolysis in hypoxic hepatoma cells by modulating the ubiquitination of HIF-1α, we treated hypoxic hepatoma cell lines Bel-7404 and Huh7 with different concentrations of CK (20, 40, and 60 μM). The results showed that protein expression levels of HIF-1α were significantly decreased after treatment with 40 and 60 μM CK (Figure 3A). The IF results showed that as the concentration of CK increased, the expression of HIF-1α in the nucleus and cytoplasm decreased (Figure 3B). To investigate the effects of CK on HIF-1α ubiquitination, the expression levels of HIF-1α ubiquitination-related proteins HSP70, HSP90, RACK1, PHD, and pVHL were detected by western blotting. The results showed that the expression levels of HSP70, HSP90, and pVHL were significantly decreased, whereas those of PHD and RACK1 were significantly increased, following treatment with different concentrations of CK (Figure 3C).

### CK Inhibited Glycolysis in Hypoxic Liver Cancer Cells

**FIGURE 4:**
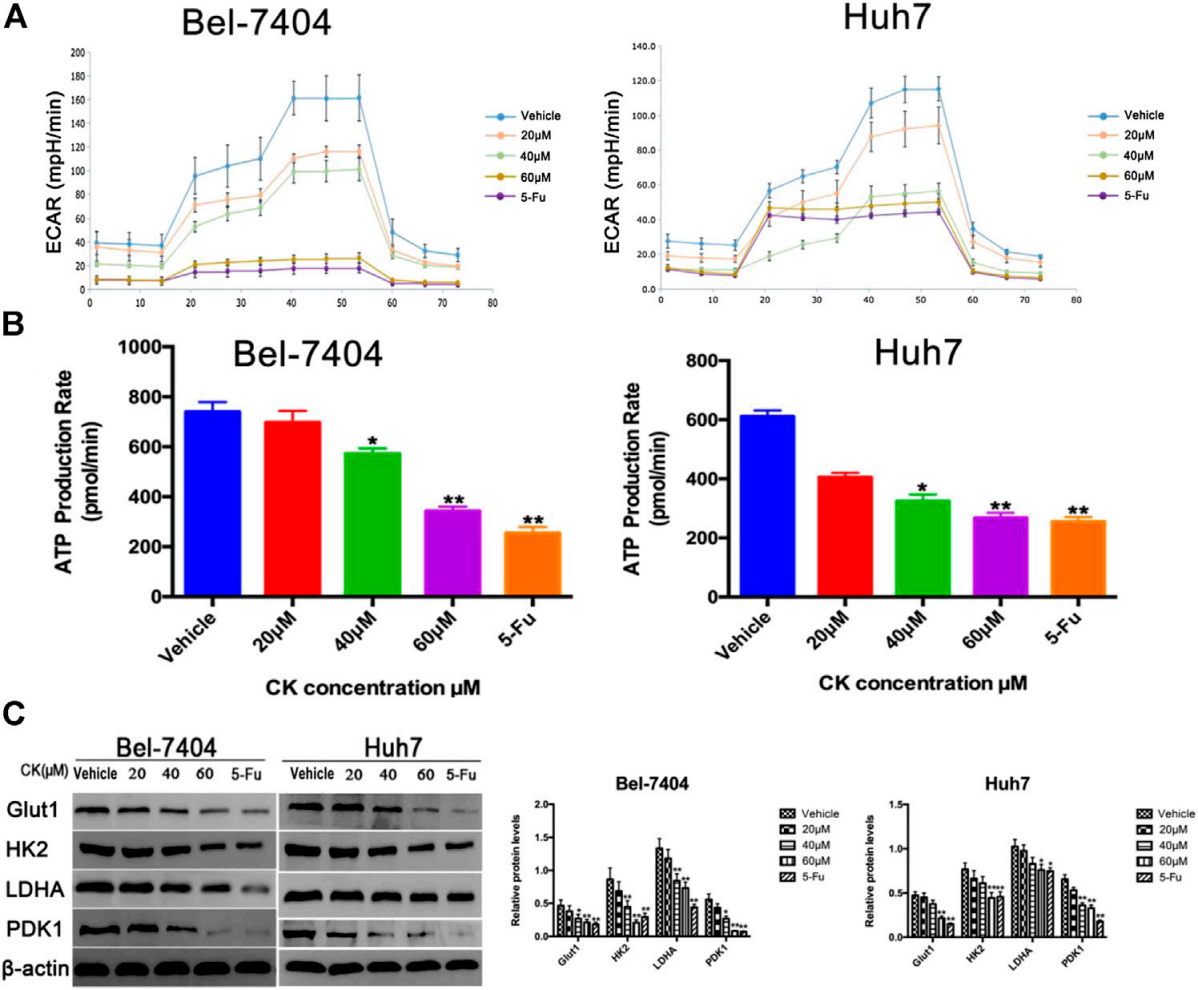
CK inhibited glycolysis in hypoxic liver cancer cells. Under hypoxic conditions, Bel-7404 and Huh7 cells were treated with different concentrations of CK (0, 20, 40, and 60 μM) for 48 h. **(A**, **B)** A metabolometer was used to measure the glycolytic rate and the rate of ATP production. **(C)** Western blot detection of the key glycolytic enzymes Glut1, HK2, LDHA, and PDK1 along with quantitative analysis compared with the vehicle group, Protein levels in the CK and 5-Fu groups were significantly reduced; data are averaged values ±standard deviation; *p < 0.05 and **p < 0.01.

To determine the effects of CK on glycolysis in hypoxic liver cancer cells, different doses (20, 40, and 60 μM) of CK were used to treat hepatoma cells under hypoxic conditions and we tested the glycolytic stress and real-time ATP production induced in Bel-7404 and Huh7 cells. The results showed that as the CK concentration increased, glycolysis and real-time ATP production decreased in a dose-dependent manner (Figures 4A, B). The expression of key glycolytic enzymes (GLUT1, HK2, LDHA, and PDK1) were detected by western blotting. Compared with the vehicle control group, GLUT1, HK2, LDHA, and PDK1 protein expression levels significantly decreased with increasing CK concentration (Figure 4C). These results suggested that CK inhibited glycolysis in Bel-7404 and Huh7 cells under hypoxic conditions.

### Bclaf1 Knockout Promoted CK-Activated HIF-1α Ubiquitination and Degradation and Inhibited Glycolysis in Hypoxic Hepatoma Cells

**FIGURE 5:**
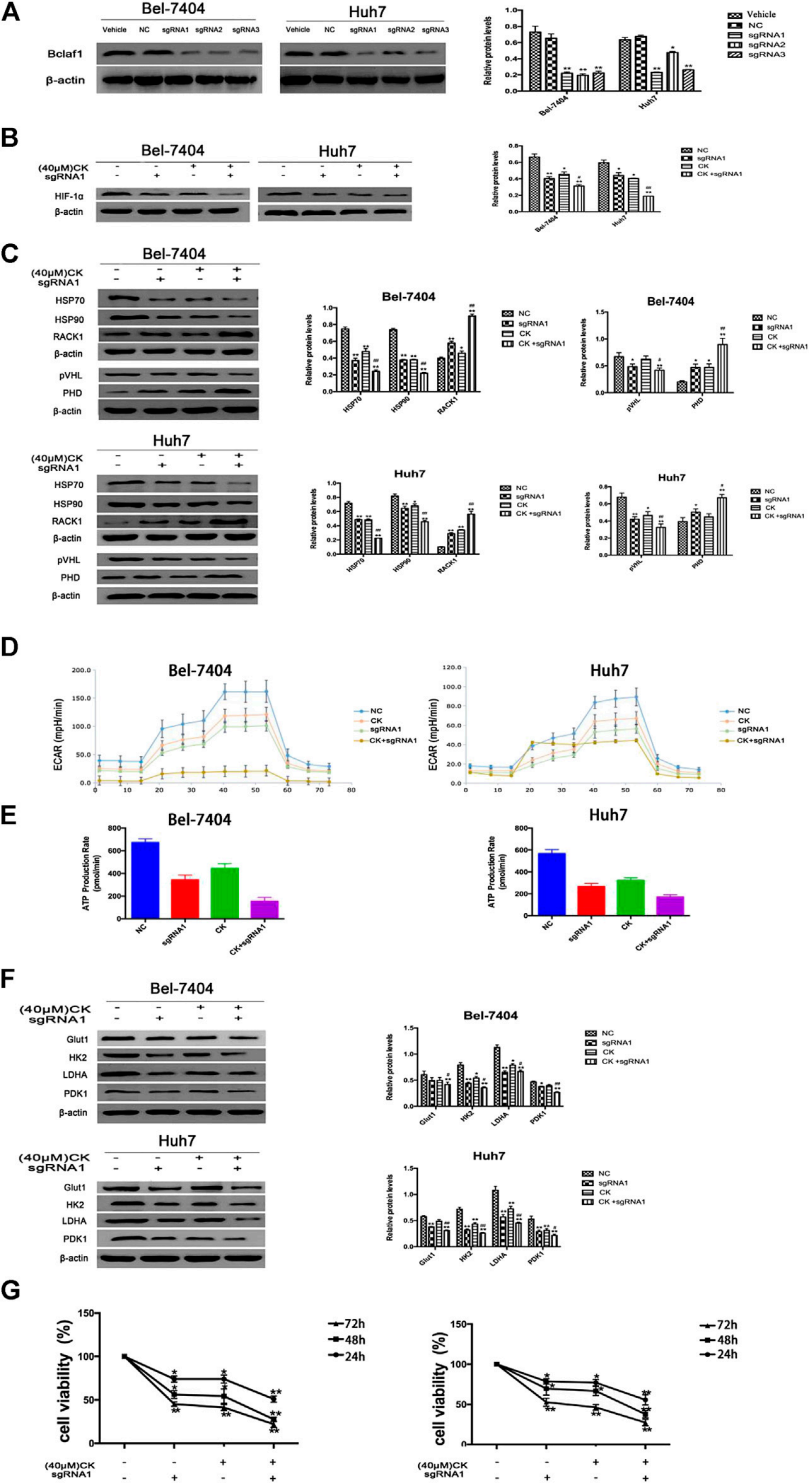
Knockout of Bclaf1 promotes CK activation of hypoxic liver cancer cell HIF-1α ubiquitination degradation and inhibits glycolysis pathway **(A)** Knocking out Bclaf1 in Bel-7404 and Huh7 cells using CRISPR/Cas9 technology. Western blot detected the expression and quantitative analysis of Bclaf1 after knockout. Compared with Vehicle group, *p < 0.05, **p < 0.01. Under hypoxic conditions, Bel-7404, Huh7, and Bclaf1-sgRNA1 transfected cells were treated with CK: **(B**, **C)** The cell glycolysis pressure and real-time ATP were detected with a bioenergy metabolic instrument; **(D)** Western blot Detection of protein expression and quantitative analysis of key glycolytic enzymes Glut1, HK2, LDHA, PDK1; **(E)** Western blot detection of HIF-1α expression and quantitative analysis; **(F)** Western blot detection of ubiquitination-related proteins (HSP70, HSP90, RACK1) and (pVHL, PHD) expression and quantitative analysis, compared with vehicle, *p < 0.05, **p < 0.01. **(G)** CCK measures the viability of both cells.

**FIGURE 6:**
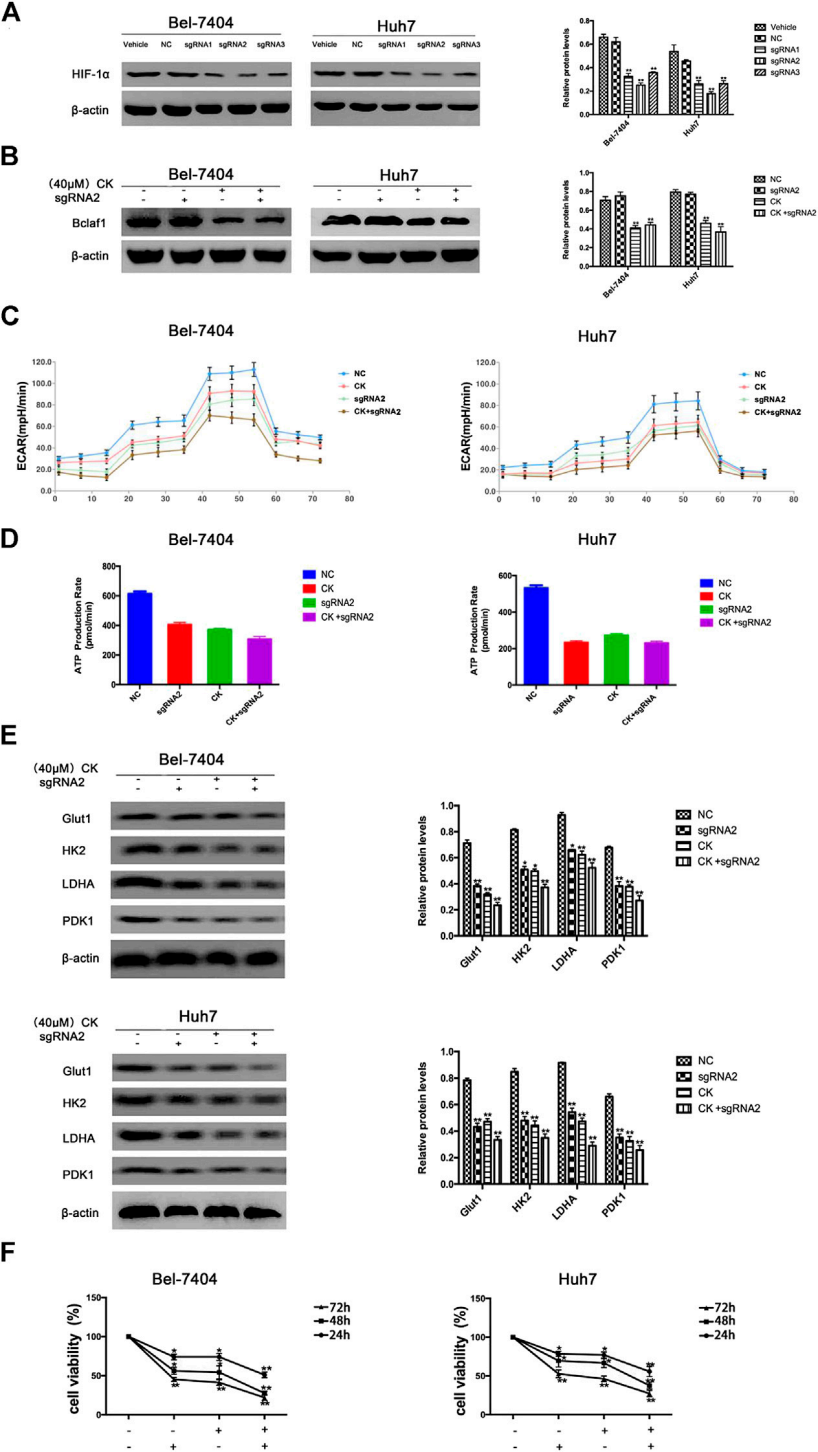
Expression of Bclaf1 after knocking out HIF-1α and the glycolysis pathway that promotes CK to inhibit proliferation **(A)** knocked out HIF-1α in Bel-7404 and Huh7 cells using CRISPR/Cas9 technology. Western blot detected the expression and quantitative analysis of HIF-1α after knockout. The data are expressed as mean ± standard deviation, compared with Vehicle group, *p < 0.05, **p < 0.01. Under hypoxic conditions, Bel-7404 and Huh7, Bclaf1-sgRNA1 transfected cells were treated with CK, respectively, **(B)** Western blot detection of HIF-1α expression and quantitative analysis; **(C**, **D)** Detect the glycolysis pressure and real-time ATP situation of the cell with a bioenergy metabolic instrument; **(E)** Western blot detection of the glycolytic key enzymes Glut1, HK2, LDHA, PDK1 protein expression and quantitative analysis, *p < 0.05, **p < 0.01. **(F)** CCK was used to detect the viability of the two cells.

In order to further verify whether the CK regulation of the HIF-1α ubiquitination degradation-mediated glycolysis pathway in hypoxic hepatocellular carcinoma involved Bclaf1, we used CRISPR/Cas9 technology to knock out Bclaf1 expression (Figure 5A), and then evaluated the expression of HIF-1α and its ubiquitination-related proteins after CK treatment. Compared with the CK+sgRNA1 group, the expression levels of HIF-1α in the Bclaf1-knockout group were reduced (Figure 5B), as were those of HSP70, HSP90, and pVHL, whereas the expression of PHD and RACK1 increased. (Figure 5C). We also examined the effects on glycolysis pressure and ATP generation rate in two types of liver cancer cells under hypoxic conditions. Compared with the CK+sgRNA1 group, the Bclaf1-knockout group showed an inhibition of glycolysis pressure and ATP generation (Figures 5D, E). Western blotting results showed that Glut1, HK2, LDHA, and PDK1 expression levels were also further reduced compared with the CK-treated group (*p < 0.05, **p < 0.01) (Figure 5F). The CCK8 assay results showed that knocking out Bclaf1 enhanced the ability of CK to inhibit the viability of hypoxic liver cancer cells (Figure 5G). In addition, we knocked out HIF-1α (Figure 6A) and found no significant differences in the protein expression of Bclaf1 after CK treatment in both types of hypoxic liver cancer cells (Figure 6B). Compared with the CK+sgRNA1 group, the Bclaf1-knockout group showed reduced glycolysis pressure and ATP generation (Figures 6C, D). Western blotting results showed that Glut1, HK2, LDHA, and PDK1 expression increased (Figure 6E) and the CCK8 assay showed that knocking out Bclaf1 promoted the reduction by CK of hypoxic liver cancer cell viability (Figure 6F). Notably, the expression of key glycolytic proteins and the CCK8 assay results after HIF-1α knockout were consistent with those obtained after knocking out Bclaf1. These results suggested that CK promoted the ubiquitination of HIF-1α in hypoxic liver cancer cells, and that the glycolysis pathway that inhibits proliferation may be mediated by HIF-1α regulation of Bclaf1, with Bclaf1 knockout further promoting the action of CK.

### CK Inhibited the Growth of Rat Primary Hepatocellular Carcinoma and the Glycolytic Pathway Regulated by Bclaf1 *In Vivo*

**FIGURE 7:**
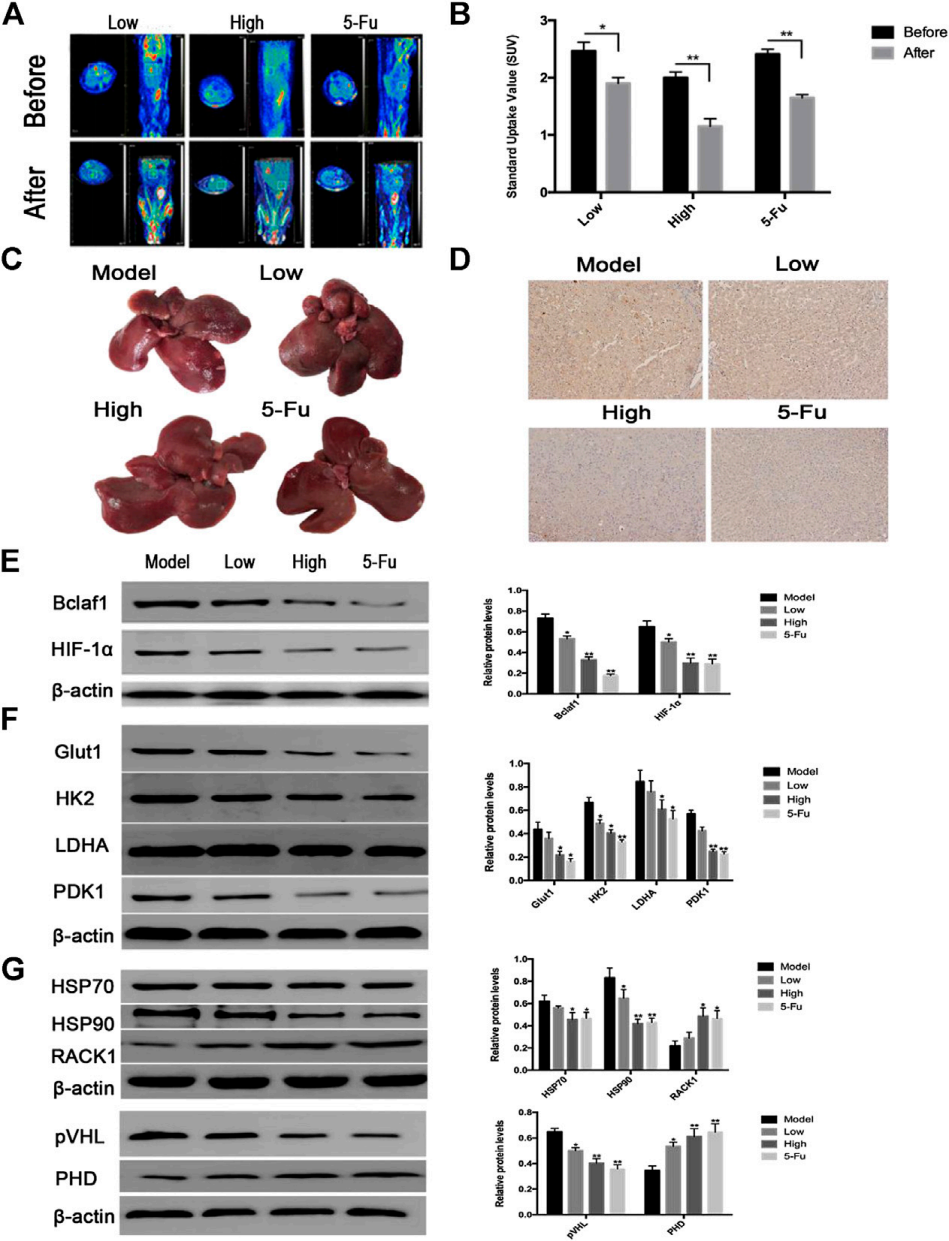
CK inhibited rat primary liver cancer tissue growth and glycolysis regulated by Bclaf1. **(A)** PET-CT was used to detect changes in tumor size in rats after CK administration. **(B)** SUV values of rat primary liver cancer before and after CK administration. **(C)** Gross view of rat liver tissue after CK administration. **(D)** The expression of Bclaf1 in rat tissues was observed by IHC after administration of CK (mag: 200×). **(E)** Western blot detection and quantitative analysis of Bclaf1 and HIF-1α after CK administration. **(F**, **G)** Western blot was used to detect the glycolytic proteins GLUT1, HK2, LDHA, and PDK1 as well as the HIF-1α ubiquitination–degradation-related proteins HSP70, HSP90, RACK1, pVHL, and PHD after administration of CK. A quantitative analysis was performed for all proteins. All data are expressed as mean ± standard deviation. Compared with vehicle, *p < 0.05, **p < 0.01.

After successfully establishing a rat model of primary liver cancer using DEN, the rats were treated with CK (2.5 mg/kg in the low-dose group; 5 mg/kg in the high-dose group), and 5-Fu (5 mg/kg in the positive control group). PET-CT results revealed tumor lesions in the liver tissue of the model group. The tumor area of rat liver tissue was reduced after CK administration compared with the model group (Figure 7A), and the SUV also changed significantly (Figure 7B). The liver tissue of the control group had a smooth surface, bright color, soft texture, and sharp edges. The liver tissue of the DEN-treated group was swollen and dull in color with a hard texture, the edges were serrated, the surface was rough, and presented grayish-white regions of different sizes. Compared with the DEN-treated group, as the dose of ginsenoside CK increased, the color of the liver tissue of rats became relatively brighter, the edges became relatively neater, the number of nodules gradually decreased, and the number of bleeding points decreased. In addition, compared with the DEN-treated group, the number of surface nodules in the positive control group (5-Fu) was reduced (Figure 7C). The IHC results showed that expression levels of Bclaf1 gradually decreased with increasing CK dose (Figure 7D), and western blotting results showed that expression levels of Bclaf1 and HIF-1α gradually decreased (Figure 7E). In addition, western blotting was used to detect the expression of key enzymes involved in glycolysis. The results showed that compared with the model group, GLUT1, HK2, LDHA, and PDK1 protein expression levels significantly decreased with increasing CK concentration (Figure 7F). Western blotting was also used to detect the expression of HIF-1α ubiquitin-related proteins. The results showed that with increasing drug concentrations, the protein expression of HSP70, HSP90, and pVHL significantly decreased, whereas expression of PHD and RACK1 significantly increased (Figure 7G). These results suggested that CK could inhibit the expression of Bclaf1 in a rat primary liver cancer model and could activate the ubiquitination of HIF-1α to inhibit glycolysis, thereby inhibiting the proliferation of liver cancer cells *in vivo*. These results were consistent with the results of our *in vitro* experiments.

## Discussion

The “Warburg effect” describes the observation that even under aerobic conditions, tumor cells prefer glycolysis for glucose metabolism. Numerous studies have confirmed that enhanced glycolysis is closely associated with the occurrence and progression of tumors and has a role in malignant progression (Burns and Manda, 2017; Yu et al., 2017). This study confirmed that under hypoxic conditions, ginsenoside CK inhibited the proliferation and glycolysis of human hepatoma cells. Likewise, the CCK8 test results showed that under hypoxic conditions, CK could inhibit liver cancer Bel-7404 and Huh7 cell viability, with cell growth blocked in the G0/G1 phase. Next, we explored whether the mechanism through which CK inhibited hypoxic liver cancer cell proliferation was related to the glycolysis pathway. The results confirmed that CK inhibited the glycolysis pathway in Bel-7404 and Huh7 cells compared with the vehicle-treated control group, suggesting that inhibition by CK of liver cancer cell proliferation under hypoxic conditions was related to the glycolysis pathway. Metabolic analysis showed that under hypoxic conditions, CK significantly reduced the extracellular acidification rate (ECAR) levels and ATP production in cells. Therefore, we speculated that CK would partially transform glucose metabolism from glycolysis to aerobic respiration in hypoxic liver cancer cells. Studies of glucose metabolites further suggested that CK reduced extracellular acidification in hypoxic liver cancer cells. The western blot results showed that CK inhibited the activities of Glut1, HK2, LDHA, and PDK1, and specifically, under hypoxic conditions, CK reduced the expression of GLUT1 in the cell membrane. It is speculated that the decrease in glucose uptake in liver cancer cells is due to downregulation of GLUT1 and the consequent reduction in its migration caused by CK. Overall, our results showed that CK reduced the expression of PDK1 and HK2, inhibited extracellular acidification, and inhibited the uptake of glucose by liver cancer cells. At the same time, CK inhibited the transport of LDHA to mitochondria, reducing the rate of glycolysis. Therefore, our results demonstrated that CK could inhibit the glycolysis pathway of hypoxic liver cancer cells.

HIF-1α, as a key transcription factor in tumor cell energy metabolism, activates many genes related to tumor metabolism and glucose transporters, and promotes the proliferation of tumor cells (Zhong et al., 1999; Ryan et al., 2000). The results of this study showed that CK inhibited the activity of key glycolytic proteins affected by HIF-1α. Therefore, we further explored whether CK could have an effect on the glycolysis of hypoxic liver cancer cells by regulating HIF-1α activity. Ubiquitination, a post-translational modification, plays a major role in the degradation of HIF-1α (Lee et al., 2019). After treatment with CK, we found that the expression of PHD increased in liver cancer cells exposed to hypoxic conditions, with a corresponding increase in the level of hydroxylation of HIF-1α. After pVHL ubiquitination, the degradation of HIF-1α was promoted. Further, we found that the expression of HSP70 also increased, suggesting that CK promoted the recruitment of the ubiquitin ligase CHIP by HSP70 and increased their interaction, thereby promoting the ubiquitination degradation of HIF-1α. In addition, HSP90 participated in stabilizing HIF-1α, whereas CK inhibited HSP90 and promoted the expression of RACK1, further confirming that RACK1 and HSP90 can competitively bind to HIF-1α, and that the increased binding of RACK1 to HIF-1α disrupts the stability of HIF-1α and promotes ubiquitination degradation of HIF-1α. Therefore, these results indicate that by inhibiting the ubiquitination degradation of HIF-1α, CK could inhibit the glycolysis pathway of hypoxic liver cancer cell proliferation.

Recent studies using RNA sequencing have detected a high expression of Bclaf1 in liver cancer Huh7 cells (Zhou et al., 2019). In our previous study using iTRAQ technology, we found that Bclaf1 was highly expressed in human liver cancer tissue cells. Thank you very much for your suggestions. According to the reviewer’s prompts, we have added the relevant discussion content as follows: At present, the mechanism of interaction between Bclaf1 and HIF-1α is not very clear. A document reported that Bclaf1 is a direct transcriptional target of HIF-1α. However, some literature (Savage et al., 2014; Zhou et al., 2019) pointed out that Bclaf1 promotes the transcription of HIF-1α in hypoxic liver cancer cells through the bZIP domain, thereby increasing the transcription of downstream targeting factors of HIF-1α and promoting the proliferation of HCC cells. After knocking out the Bclaf1 gene, HIF-1α levels were significantly reduced. It shows that Bclaf1 is the upstream regulator of HIF-1α in hypoxic microenvironment. In order to further clarify the interaction mechanism between Bclaf1 and HIF-1α, we use CRISPR/Cas9 technology to knock out Bclaf1, and detect the expression of HIF-1α in liver cancer cells after knocking out Bclaf1 under hypoxia. The results of the study were similar to Ying et al. The expression of HIF-1α in liver cancer cells was significantly decreased after Bclaf1 was knocked out. It can be speculated that Bclaf1 acts as an upstream regulator of HIF-1α in the hypoxia microenvironment. We thus investigated whether CK could inhibit the expression of HIF-1α through Bclaf1 and thus affect the glycolysis pathway of liver cancer cells. Western blotting results showed that CK effectively inhibited the expression of Bclaf1 and HIF-1α, and Co-IP results showed that CK significantly inhibited the binding of Bclaf1 and HIF-1α. In order to determine the role of Bclaf1 in the inhibition of liver cancer cell proliferation by CK, we knocked out Bclaf1 and found that it enhanced the ability of CK to activate HIF-1α ubiquitination and inhibit the HIF-1α-mediated glycolysis pathway to inhibit proliferation. In addition, we knocked out HIF-1α and observed that there was no significant difference in the expression of Bclaf1 following treatment with CK, whereas knockout of HIF-1α further promoted the proliferative glycolysis pathway induce by CK inhibition. Therefore, we speculate that the inhibition of liver cancer cell proliferation by CK may involve regulation of Bclaf1, which in turn inhibits the HIF-1α-mediated glycolysis pathway.

Finally, we used a DEN-induced primary liver cancer rat model to analyze the effects of CK treatment *in vivo*. We used Bin et al.’s experimental method (Xiao et al., 2014) to model and treat primary liver cancer in rats. The results showed that CK administration exerted a certain inhibitory effect on the growth of primary liver cancer in rats. PET/CT scans revealed tumors were smaller after treatment and the SUV value decreased, suggesting that CK inhibited the glucose intake in the primary liver cancer rat model. The IHC results of tumor tissue showed that CK inhibited Bclaf1 expression in a dose-dependent manner in the DEN-rat model. Western blot results confirmed that the expression of HIF-1α was reduced and the expression of ubiquitination-related proteins and glycolysis-related proteins was consistent with those of the experimental results *in vitro*.

**FIGURE 8:**
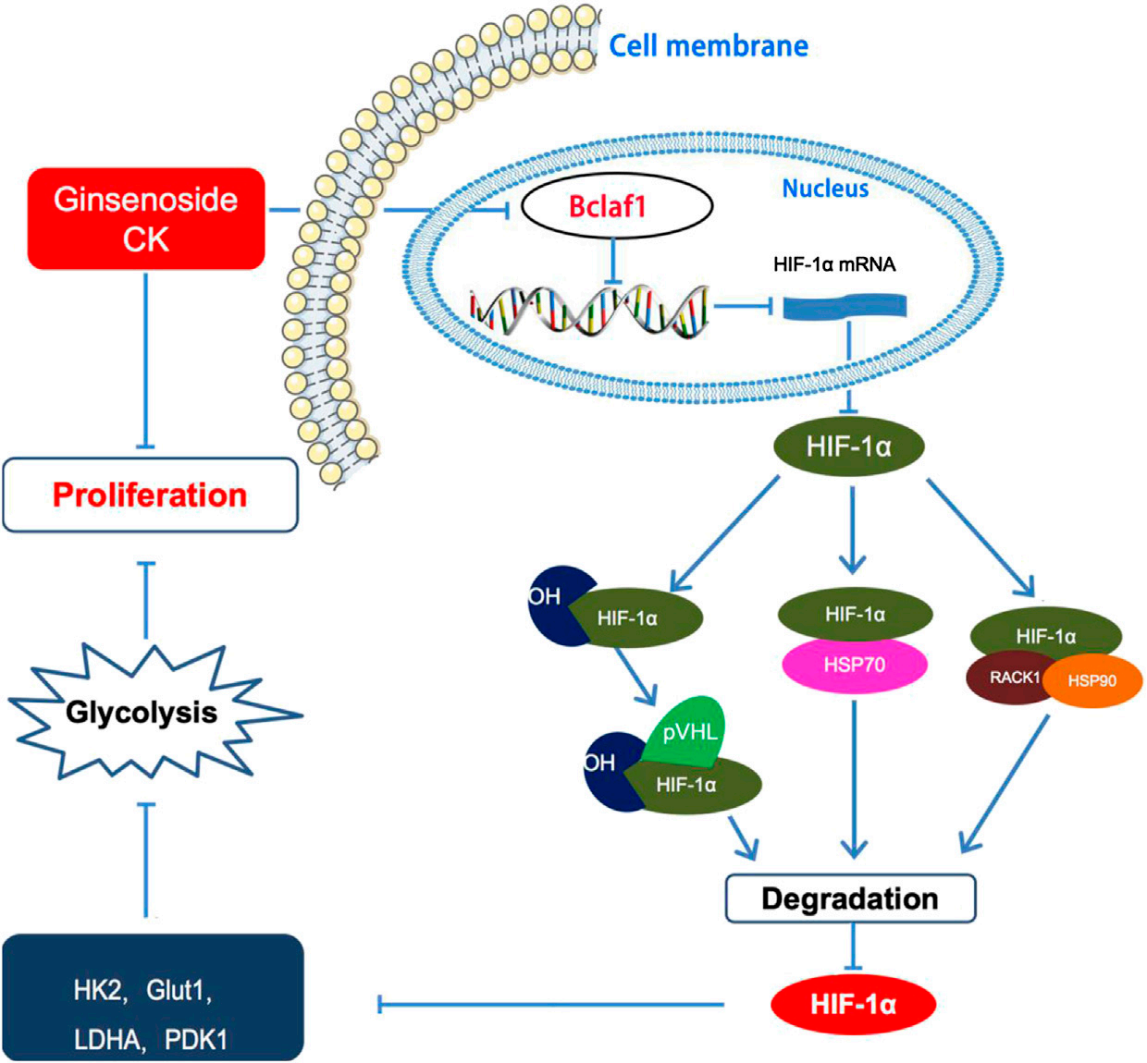
Ginsenoside compound K regulates HIF-1α-mediated glycolysis through Bclaf1 to inhibit the proliferation of human liver cancer cells.

Overall, our study showed that CK inhibits the proliferation of hypoxic liver cancer cells (Bel-7404 and Huh7) and exerts its anti-tumor function by regulating the expression of Bclaf1. CK inhibits the expression of HIF-1α and inhibits HIF-1α-mediated glycolysis in hypoxic Bel-7404 and Huh7 cells, thereby inhibiting their proliferation (Figure 8). As hypoxia is a key condition that limits the growth of solid tumors (Harris, 2002; Vaupel et al., 2004), CK treatment thus may play a vital role in inhibiting the progression of liver cancer. Therefore, our study provides a novel approach for the clinical treatment of liver cancer using derivatives from TCM. Specifically we provide mechanistic evidence supporting the use of CK to target Bclaf1 as a potential drug for the treatment of liver cancer.

## Conclusion

Our study provides evidence that under hypoxic conditions CK inhibited the expression of Bclaf1 in Bel-7404 and Huh7 cells, promoted the degradation of HIF-1α ubiquitination, and inhibited the HIF-1α-mediated cellular glycolysis pathway to inhibit the proliferation of liver cancer cells *in vitro*. These results are supported by *in vivo* experiments in the DEN-induced rat model, whereby CK inhibited the growth of primary liver cancer, down-regulated the expression of Bclaf1 and HIF-1α, promoted the ubiquitination degradation of HIF-1α, and inhibited glycolysis. In conclusion, this study provides strong evidence that CK may exert therapeutic effects by inhibiting the HIF-1α-mediated glycolysis pathway by downregulating the expression of Bclaf1 in liver cancer cells, and thereby inhibiting cell proliferation and cancer progression in the hypoxic microenvironment observed in liver cancer.

## Data Availability Statement

The original contributions presented in the study are included in the article, further inquiries can be directed to the corresponding author.

## Ethics Statement

The experiment procedures were approved by the Institutional Animal Care and Use Committee of Yanbian University (Resolution number, 201501022).

## Author Contributions

XZ and SZ designed the study; SZ and MZ performed the research, analyzed data, and wrote the initial draft of the paper. The remaining authors contributed to refining the ideas, carrying out additional analyses and finalizing this paper.

## Funding

This study was supported by the National Natural Science Foundation of China (81760728) and Jilin Province Science and Technology Development Plan Project (20200201526JC).

## Conflict of Interest

The authors declare that the research was conducted in the absence of any commercial or financial relationships that could be construed as a potential conflict of interest.
